# Exercise-Induced Rhabdomyolysis With Supplements (Creatine, β-Alanine, Citrulline Malate, and β-Ecdysterone)

**DOI:** 10.7759/cureus.104852

**Published:** 2026-03-08

**Authors:** Patrycja Chowaniec, Milosz Miedziaszczyk, Martyna Kucia, Mateusz Konieczny, Ilona Idasiak-Piechocka

**Affiliations:** 1 Department of General and Transplant Surgery, Poznan University of Medical Sciences, Poznan, POL; 2 Department of Clinical Pharmacy and Biopharmacy, Poznan University of Medical Sciences, Poznan, POL

**Keywords:** beta-alanine, beta-ecdysterone, citrulline, creatine supplement, exercise-induced rhabdomyolysis

## Abstract

Dietary supplements such as creatine, β-alanine, citrulline malate (CM), phosphatidic acid (PA), and β-ecdysterone are widely used to enhance exercise performance and muscle hypertrophy. Although generally considered safe, their combined use with high-intensity training may increase the risk of exertional rhabdomyolysis and secondary liver or kidney injury. We report the case of a 23-year-old physically active man admitted with severe myalgia, muscle weakness, fever, and dark brown urine following a prolonged (>4 hours) resistance training session. The patient regularly used multiple dietary supplements, including creatine (5 g), β-alanine, CM (10 g), PA (1.5 g), β-ecdysterone, protein, vitamins, and other micronutrients. Laboratory evaluation revealed markedly elevated creatine kinase (>120,000 U/L) and increased aminotransferases (aspartate aminotransferase (AST) 1,275 U/L; alanine aminotransferase (ALT) 337 U/L), with preserved renal function (creatinine 1.0 mg/dL; estimated glomerular filtration rate (eGFR) 108 mL/min/1.73 m²). No electrolyte disturbances or cardiac injury were observed. The patient received intensive intravenous fluid therapy, resulting in gradual clinical and biochemical improvement over 10 days without development of acute kidney injury (AKI). Creatine and β-alanine enhance exercise capacity through complementary mechanisms involving adenosine triphosphate (ATP) resynthesis and intracellular buffering, potentially enabling greater training loads and increasing risk of muscle injury. The ergogenic efficacy of CM remains inconclusive, while PA and β-ecdysterone may stimulate anabolic signaling via mTOR-related and estrogen receptor-mediated pathways, respectively. Although β-ecdysterone has not previously been linked to rhabdomyolysis, its metabolic effects may theoretically exacerbate muscle damage after extreme exertion. Genetic predisposition, including LPIN1 variants, cannot be excluded and represents a limitation of our study, as genetic testing was not performed. This case highlights the potential risk of severe rhabdomyolysis associated with intensive exercise combined with multi-ingredient supplementation. Healthcare professionals should inquire about supplement use in patients presenting with muscle injury. Early recognition and prompt intravenous hydration remain essential to prevent renal complications. Further studies are needed to clarify the safety profile of emerging anabolic supplements, particularly β-ecdysterone.

## Introduction

Among individuals who regularly engage in physical training, the use of dietary supplements is commonly reported as a means of supporting exercise adaptation, improving physical performance, and increasing muscle mass and strength. The most frequently used products include creatine, protein supplements, and vitamins. The described adverse effects of these supplements include rhabdomyolysis, gastrointestinal disorders, and exercise-induced acute kidney injury (AKI) [1].

Rhabdomyolysis occurs as a result of the breakdown of the muscle cell membrane, leading to cell necrosis. The cause is considered to be either a defect of the cell membrane or a dysfunction of the sodium-potassium pump, which is indirectly responsible for the influx of calcium into the cell interior. As a consequence, cell necrosis develops [2]. As a result of necrosis, potentially toxic intracellular substances, such as creatine kinase (CK), myoglobin, amino acids, and electrolytes, are released into the plasma. The concentration of myoglobin in the blood exceeds the binding capacity of haptoglobin. Unbound myoglobin is filtered in the glomeruli and then accumulates in the tubular lumen, causing nephrotoxicity and renal tubular obstruction, ultimately leading to AKI [3,4]. The objective of this article is to present a case report of rhabdomyolysis with liver damage resulting from supplement-dependent muscle overload. All procedures adhered to the guidelines outlined in the Declaration of Helsinki. Informed consent was obtained from the patient.

## Case presentation

A 23-year-old patient was admitted to the Department of Internal Medicine due to rhabdomyolysis. The patient reported severe muscle pain and dark brown discoloration of the urine. He described the pain as burning and dull in character, along with muscle tenderness, muscle weakness, and fever. On the morning three days before hospital admission, he had engaged in prolonged physical exercise at the gym, lasting more than four hours. The patient is regularly physically active, training twice a week for about one hour. His medical history included depression treated with vortioxetine. Daily, he takes the following preparations: ashwagandha, coenzyme Q10, vitamin C, vitamin D3, vitamin B12, vitamin K2, vitamin B2, magnesium, chromium, zinc, lutein, omega-3, and protein (casein) 30-60 g. On training days, he takes phosphatidic acid (PA) 1.5 g, creatine 5 g, beta-alanine, and citrulline malate (CM) 10 g before training and beta-ecdysterone after the training.

On admission, vesicular breath sounds over the lungs; chest X-ray and abdominal ultrasound examination showed no abnormalities. Heart rate was 70 beats per minute with respiratory arrhythmia. Physical examination was otherwise unremarkable. Initial laboratory tests revealed elevated creatine kinase (CK) (>120,000 U/L) and aminotransferases: alanine aminotransferase (ALT) 337 U/L, aspartate aminotransferase (AST) 1.275 U/L, potassium 4.1 mmol/L, creatinine 1.00 mg/dL, and glomerular filtration rate (GFR) (CKD-EPI) 108 mL/min/1.73 m². Due to rapid hospitalization and treatment, AKI was not observed. Renal filtration function remained preserved. We did not observe any deviations in the morphology examination (hemoglobin (HGB) 16.5 g/dL, erythrocytes (RBC) 5.3 x 106/uL, leukocytes (WBC) 6.2 x 103/uL), general urine test without any abnormalities, other parameters: C-reactive protein (CRP) 7.7 mg/dL, sodium 134 mmoL/L, potassium 4.1 mmoL/L, magnesium 0.85 mmoL/L, calcium 2.4 mmoL/L, and troponin < 1.5 ng/L (Table 1). The laboratory test results were within normal reference ranges. The patient was treated with a multi-electrolyte fluid in a slow drip infusion (2.0 L/24 h) and water p.o. (1.0 L/24 h), achieving a daily diuresis of 2.5 L. As a result of the implemented treatment, after 10 days, an improvement in laboratory parameters and in the patient’s general condition was achieved. Table 2 shows the relationship between changes in selected laboratory parameters and the duration of hospitalization.

**Table 1:** Laboratory parameters results upon patient admission to hospital. Abbreviations: GFR CKD-EPI: glomerular filtration rate (eGFR) Chronic Kidney Disease Epidemiology Collaboration; HGB: hemoglobin; RBC: red blood cell; HCT: hematocrit; WBC: white blood cell; NEU: neutrophils; LYM: lymphocytes; MONO: monocytes; EOS: eosinophils; BASO: basophils; MCV: mean corpuscular volume; MCH: mean corpuscular hemoglobin; MCHC: mean corpuscular hemoglobin concentration; RDW: red cell distribution width; PLT: platelet; MPV: mean platelet volume; CRP: C-reactive protein

Parameter	Day 1 value	Reference range
Creatine mg/dL	1	<1.2 mg/dL
GFR CKD-EPI mL/min/1.73 m²	108	>90 mL/min/1.73 m²
HGB g/dL	16.5	13.5-18.0 g/dL
RBC 10^6^/uL	5.30	4.6-6.5 10^6^/uL
HCT %	49.7	40.0-52.0%
WBC 10^3^/uL	6.20	4.0-10.0 10^3^/uL
NEU 10^3^/uL	3.5	1.8-7.8 10^3^/uL
LYM 10^3^/uL	2.3	1.0-3.0 10^3^/uL
MONO 10^3^/uL	0.4	0.3-1.0 10^3^/uL
EOS 10^3^/uL	0.1	0.0-0.5 10^3^/uL
BASO 10^3^/uL	0.01	0.0-0.1 10^3^/uL
MCV fL	93.7	80.0-98.0 fL
MCH pg	31.1	27.0-32.0 pg
MCHC g/dL	33.2	31.0-37.0 g/dL
RDW %	13.5	11.5-14.5%
PLT 10^3^/uL	202	150-400 10^3^/uL
MPV fL	8.7	7.0-12.0 fL
CRP mg/dL	7.70	<10.0 mg/dL
Na mmol/L	134	136-145 mmol/L
K mmol/L	4.10	3.5-5.1 mmol/L
Mg mmol/L	0.85	0.7-1.05 mmol/L
Ca mmol/L	2.40	2.1-2.5 mmol/L
Troponin ng/L	<1.50	<13 ng/L

**Table 2:** Relationship between changes in selected laboratory parameters and the duration of hospitalization. Abbreviations: CK: creatine kinase; ALT: alanine aminotransferase; AST: aspartate aminotransferase

Day	CK (U/L)	ALT (U/L)	AST (U/L)
Day 1	>120,000	337	1275
Day 2	94,433	328	939
Day 3	79,423	-	-
Day 4	30,007	261	374
Day 5	7,204	251	174
Day 7	814	165	44.2
Day 10	238	91	18.6
Reference range	55-170 U/L	<50 U/L	17-59 U/L

## Discussion

The characteristics of resistance training involve the manipulation of parameters such as training session frequency, volume (number of sets and repetitions), load, and movement velocity. In practice, this entails performing exercises targeting different muscle groups in sets with a specified number of repetitions, at intensities approaching maximal muscular capacity, often to the point of muscular fatigue. A key aspect of resistance training is the substantial contribution of anaerobic metabolism, both glycolytic and phosphagen systems. High-intensity exercises of short to moderate duration require significant anaerobic energy production. Anaerobic metabolism enables the generation of high power output in a short time, and its contribution to total energy expenditure can exceed 40%, particularly during sets performed to muscular failure [5]. In one study, the contributions of aerobic and anaerobic metabolism during resistance exercise at 80% 1-RM (one-repetition maximum) were assessed in 14 regularly trained men. Anaerobic metabolism was estimated using the accumulated oxygen deficit (AOD) method, while aerobic metabolism was determined based on oxygen uptake (VO₂). The results demonstrated that at 80% 1-RM, anaerobic metabolism predominated, particularly in exercises involving large muscle mass, such as the half squat, highlighting the key role of anaerobic energy pathways in short-term, high-intensity resistance efforts [6].

Our patient performed resistance (strength) training aimed at increasing muscular strength and mass. The program included isolated exercises (e.g., biceps curls), as well as compound exercises engaging larger muscle groups (squats, deadlifts, and bench press), executed in sets with a predetermined number of repetitions and individually tailored loads. Rest intervals between sets allowed for recovery, while controlled movement velocity ensured safety and training efficiency. The high intensity of the training engaged anaerobic metabolism, supporting power generation, muscular adaptations, and increased energy expenditure.

The differential diagnosis of elevated CK levels primarily includes inflammatory myopathies, muscular dystrophies, and thyroid dysfunction. In inflammatory myopathies, symptoms develop subacutely over several weeks and are often accompanied by muscle pain, whereas in rhabdomyolysis, the onset is sudden. CK levels in rhabdomyolysis usually normalize within a few days to weeks, whereas in myositis, they remain elevated until treatment is initiated. Muscular dystrophies have a chronic, progressive course lasting years, so a detailed medical and family history is particularly important. Reddish-brown urine may result from myoglobinuria or hematuria. Differentiation is based on microscopic examination of the urine sediment for the presence of erythrocytes. Similar discoloration can also be caused by certain foods or medications; however, in these cases, CK levels remain normal [7].

Creatine acts as a store and transporter of high‑energy phosphate groups, enabling the rapid resynthesis of ATP from ADP and providing the energy necessary for proper muscle function and contraction. In a person weighing about 70 kg, the daily requirement for creatine is approximately 2 g. This compound is derived both from food and from endogenous synthesis in the body [8]. Creatine is a widely used ergogenic supplement employed by athletes to improve their ability to perform short‑term, high‑intensity muscular effort, particularly by increasing the strength and power of skeletal muscles. Supplementation, most commonly in the form of creatine monohydrate, increases the concentration of creatine and phosphocreatine in skeletal muscles, which allows for faster ATP resynthesis during anaerobic efforts and delays the onset of muscle fatigue. Increased phosphocreatine stores provide a high‑energy reserve necessary for short‑duration, high‑intensity exercise [9]. Improved performance capacity makes it possible to increase the intensity and duration of training, which in turn increases the risk of exertional rhabdomyolysis [10].

The most common causes of acquired rhabdomyolysis include direct muscle injury, prolonged compression or immobilization, extreme physical exertion, alcohol, drugs of abuse, medications, and both bacterial and viral infections, as well as toxins. Congenital causes include metabolic myopathies (e.g., McArdle disease), structural myopathies, and mutations in ion channel genes [11]. Elevated aminotransferases and abnormal liver function parameters are often observed in severe rhabdomyolysis, as these enzymes may originate both from the liver and from damaged muscle fibers [12].

Supplementation with β‑alanine increases the concentration of carnosine in skeletal muscles, particularly in type II fibers, which improves acid buffering, delays neuromuscular fatigue, and shifts the anaerobic threshold. The ergogenic effects are most evident in efforts lasting 0.5-10 minutes, and the greatest benefits are observed in untrained individuals. Although short bouts of high‑intensity exercise do not increase muscle carnosine content, chronic, intensive training may lead to its elevation. In addition, carnosine may influence excitation-contraction coupling and provide protection against reactive oxygen species, suggesting that the ergogenic mechanisms of β‑alanine are multifactorial and extend beyond its buffering effect alone [13,14,15]. The physiological basis of the synergy between creatine and β‑alanine is well established: creatine supports the ATP‑PCr system, providing a rapid source of energy for short‑duration, high‑intensity efforts, whereas β‑alanine, by increasing carnosine synthesis, acts as a buffer for H⁺ ions. As a result, it delays the decline in pH associated with anaerobic glycolysis and reduces the sensation of fatigue during repeated bouts of exercise. This complementary action likely explains why the effects of β‑alanine are more apparent in repeated exercise bouts than in single maximal strength attempts [16]. In the context of our patient, the combination of these components may have contributed to intense muscular overload, increasing the risk of developing rhabdomyolysis.

The available scientific evidence on the effectiveness of CM supplementation in improving muscle strength, physical performance, and recovery is limited and inconclusive. Current meta-analyses and interventional studies indicate that in healthy, trained individuals, CM supplementation does not lead to a significant improvement in maximal strength of either the upper or lower limbs [17]. Studies involving team-sport athletes further confirm these observations. Neither a single dose of 3 g nor 6 g of CM has shown a beneficial effect on physical performance, exercise capacity, subjective perception of fatigue, or the rate of post-exercise recovery. These findings call into question the rationale for using low and moderate doses of CM as a strategy to support short-term performance in high-intensity sports [18]. On the other hand, data from systematic reviews and meta-analyses indicate that citrulline supplementation - both in the form of L-citrulline and CM - may help reduce the subjective level of fatigue (RPE) and muscle soreness after exercise. This effect has been observed mainly when the supplement is taken approximately 60 minutes before physical activity. However, it should be emphasized that the beneficial impact relates primarily to subjective measures, rather than to objective parameters of mechanical performance or maximal strength [19]. Some reports suggest that acute CM supplementation at a dose of around 8 g may inconsistently improve strength endurance; however, there is no clear evidence confirming its effectiveness in increasing muscle power, accelerating recovery, or inducing beneficial muscular adaptations. Importantly, the authors indicate that L-citrulline has a stronger and more consistent ergogenic profile than CM, which may result from differences in bioavailability and metabolic mechanisms [20].

Preclinical data obtained from animal models further highlight the complexity of CM’s effects. Short-term supplementation may increase resistance to fatigue in the initial phase of exercise and improve oxidative capacity and contraction efficiency of muscles during the first sets of exercises. These effects, however, diminish with repeated efforts, and CM supplementation does not support the restoration of high-energy phosphorylated compounds in the muscles after exercise. Consequently, at the current stage of knowledge, there is no basis for unequivocally recommending CM as effective nutritional support in training that involves repeated bouts of exercise with short rest intervals [17]. CM supplementation may increase nitric oxide (NO) production, which improves vasodilation of blood vessels in working muscles, increases blood flow and mitochondrial activity, and may potentially prolong exercise duration and delay fatigue. Although CM effectively increases systemic arginine concentrations, its action may be limited by enzymatic saturation, competition with other metabolic pathways, high arginase activity, and reduced NO synthase function. Further research is needed to determine the optimal dose, timing of administration, and mechanism of action of CM [21].

PA is a structural phospholipid of cell membranes that also functions as an intracellular signaling molecule involved in regulating the activity of signaling proteins. PA is one of the endogenous activators of the mTOR pathway, which plays a key role in controlling the rate of muscle protein synthesis. It has been shown that PA supplementation combined with resistance training may lead to increased skeletal muscle hypertrophy and gains in muscle strength [22]. The LPIN1 gene encodes the protein lipin 1, which has a bifunctional role, combining enzymatic activity with transcriptional regulatory functions to support muscle energy homeostasis. Lipin 1 acts as a PA phosphatase, catalyzing the hydrolytic conversion of PA to diacylglycerol (DAG) with the concurrent release of inorganic phosphate (Pi). DAG is a key metabolic intermediate used both in the biosynthesis of membrane phospholipids, such as phosphatidylethanolamine and phosphatidylcholine, and in the synthesis of triacylglycerol (TAG), which serves as the main form of energy storage in the cell [23,24]. Mutations in LPIN genes lead to severe metabolic phenotypes, including, among others, rhabdomyolysis (LPIN1), autoinflammatory diseases (LPIN2), and disorders of intestinal lipoprotein formation (LPIN2 and LPIN3) [25]. LPIN1 has been identified as a newly recognized cause of recurrent episodes of acute myoglobinuria in children. Consequently, mutations in LPIN1 are considered a key genetic factor in the pathogenesis of early childhood rhabdomyolysis and myoglobinuria [26]. In our patient, genetic testing was not performed, which is a limitation of our case report.

**Figure 1:**
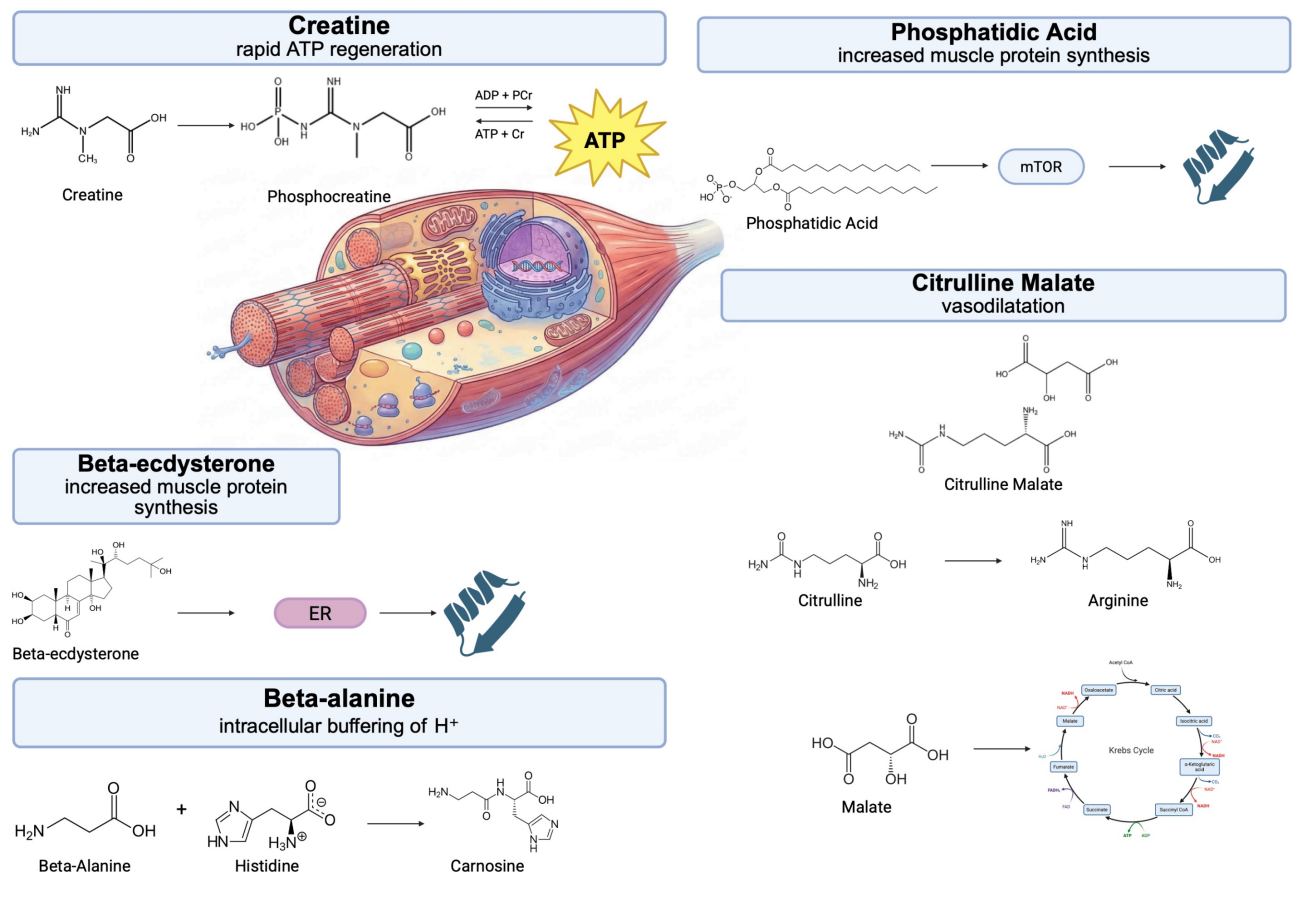
Effect of creatine, beta-ecdysterone, beta-alanine, phosphatidic acid, and citrulline malate on muscles. Abbreviations: ADP: adenosine diphosphate; ATP: adenosine triphosphate; Cr: creatinine; ER: estrogen receptor; mTOR: mammalian target of rapamycin; PCr: phosphocreatine Image created in BioRender (https://BioRender.com/6s405hm)

β-ecdysterone (20-hydroxyecdysterone) is a plant-derived steroid that exhibits strong anabolic activity both in vitro and in vivo. In contrast to classical anabolic steroids, its action is not mediated by the androgen receptor (AR) - studies indicate that β-ecdysterone does not show significant affinity for AR. Instead, the anabolic effects of β-ecdysterone are associated with the activation of estrogen receptors, with preferential involvement of the ERβ subtype. Experiments with ER-selective agonists and antagonists suggest that ERβ plays a key role in modulating β-ecdysterone-induced muscle hypertrophy [27,28]. Excessive loading or stretching of skeletal muscle beyond its physiological capacity leads to muscle damage and a transient reduction in its functional capability. Although the improvement in muscle function observed after 7 days of 20E (20-hydroxyecdysone) supplementation cannot be unequivocally attributed solely to anabolic mechanisms dependent on the PI3K/Akt/mTORC1 pathway, the available literature data suggest that 20E may serve as an anabolic stimulus for damaged muscle tissue, accelerating its regeneration. In one study, it was shown that daily administration of 20E enabled full restoration of skeletal muscle function in both adult and older mice within 7 days after damage induced by eccentric contractions [29]. It has been demonstrated that β-ecdysterone exerts a significant effect both on parameters including body mass and muscle mass, and on performance indicators, such as the maximum load achieved in the one-repetition maximum bench press test. The results obtained are supported by studies conducted in vitro [30]. Despite the growing popularity of β-ecdysterone as a dietary supplement, no cases of rhabdomyolysis associated with its use have so far been described in the available scientific literature. β-ecdysterone stimulates myocyte metabolism and protein synthesis [31]. In conditions of severe muscle damage, increased cellular energy demand can potentially lead to increased ATP deficit, increased oxidative stress, and secondary exacerbation of myocyte damage, which theoretically may promote the development of rhabdomyolysis. In the available scientific literature, there is no information regarding the toxic doses of the discussed toxic supplements. The effects of creatine, beta-ecdysterone, beta-alanine, PA, and CM on muscle are shown in Figure 1.

## Conclusions

There is a potential risk of rhabdomyolysis associated with intense physical exercise combined with multi-ingredient supplementation. The use of creatine and β-alanine may improve performance and increase the intensity and duration of training, which in turn may raise the risk of developing exertional rhabdomyolysis. The metabolic effects of β-ecdysterone may exacerbate skeletal muscle damage following extreme exercise. The lack of data on the use of β-ecdysterone indicates the need for further studies on the safety and efficacy of this compound. Both medical personnel and patients must be aware of the potential risk of serious complications associated with supplements. Early and aggressive hydration remains a crucial strategy in the treatment of rhabdomyolysis, regardless of its cause, to minimize renal complications and enhance patient outcomes.
